# Open-source, small-animal magnetic resonance-guided focused ultrasound system

**DOI:** 10.1186/s40349-016-0066-7

**Published:** 2016-09-05

**Authors:** Megan E. Poorman, Vandiver L. Chaplin, Ken Wilkens, Mary D. Dockery, Todd D. Giorgio, William A. Grissom, Charles F. Caskey

**Affiliations:** 1 Department of Biomedical Engineering, Vanderbilt University, PMB 351631 2301 Vanderbilt Place, Nashville, 37235 TN USA; 2 Institute of Imaging Science, Vanderbilt University, 1161 21st Avenue South, Nashville, 37232 TN USA; 3 Chemical and Physical Biology Program, Vanderbilt University, 1161 21st Avenue South, Nashville, 37232 TN USA; 4 Department of Radiology, Vanderbilt University, 1161 21st Avenue South, Nashville, 37232 TN USA

**Keywords:** High-intensity focused ultrasound (HIFU), MR-guided focused ultrasound (MRgFUS), Preclinical, Open source

## Abstract

**Background:**

MR-guided focused ultrasound or high-intensity focused ultrasound (MRgFUS/MRgHIFU) is a non-invasive therapeutic modality with many potential applications in areas such as cancer therapy, drug delivery, and blood-brain barrier opening. However, the large financial costs involved in developing preclinical MRgFUS systems represent a barrier to research groups interested in developing new techniques and applications. We aim to mitigate these challenges by detailing a validated, open-source preclinical MRgFUS system capable of delivering thermal and mechanical FUS in a quantifiable and repeatable manner under real-time MRI guidance.

**Methods:**

A hardware and software package was developed that includes closed-loop feedback controlled thermometry code and CAD drawings for a therapy table designed for a preclinical MRI scanner. For thermal treatments, the modular software uses a proportional integral derivative controller to maintain a precise focal temperature rise in the target given input from MR phase images obtained concurrently. The software computes the required voltage output and transmits it to a FUS transducer that is embedded in the delivery table within the magnet bore. The delivery table holds the FUS transducer, a small animal and its monitoring equipment, and a transmit/receive RF coil. The transducer is coupled to the animal via a water bath and is translatable in two dimensions from outside the magnet. The transducer is driven by a waveform generator and amplifier controlled by real-time software in Matlab. MR acoustic radiation force imaging is also implemented to confirm the position of the focus for mechanical and thermal treatments.

**Results:**

The system was validated in tissue-mimicking phantoms and in vivo during murine tumor hyperthermia treatments. Sonications were successfully controlled over a range of temperatures and thermal doses for up to 20 min with minimal temperature overshoot. MR thermometry was validated with an optical temperature probe, and focus visualization was achieved with acoustic radiation force imaging.

**Conclusions:**

We developed an MRgFUS platform for small-animal treatments that robustly delivers accurate, precise, and controllable sonications over extended time periods. This system is an open source and could increase the availability of low-cost small-animal systems to interdisciplinary researchers seeking to develop new MRgFUS applications and technology.

## Background

Focused ultrasound (FUS) is a promising non-invasive surgical modality with the ability to thermally and mechanically affect target tissue with minimal effects in intervening and surrounding tissues. It has seen development for many applications including tumor ablation and hyperthermia [1], immunotherapy [2, 3], neuromodulation [4, 5], blood-brain barrier opening [6], drug delivery [7], blood vessel clearing [8], and mechanical tissue digestion [9]. Though FUS was first explored for non-invasive surgery as far back as the 1950s, it was hindered by a lack of imaging guidance, which has been overcome with the development of magnetic resonance imaging (MRI) and its integration with FUS. MRI provides excellent soft tissue contrast and is sensitive to changes in tissue resulting from FUS treatment. Commercial clinical MR-guided FUS (MRgFUS) systems use MRI for treatment planning, treatment monitoring via real-time temperature imaging [10], and treatment assessment.

In spite of its promise, the availability of preclinical MRgFUS systems for research remains limited due to the high cost and often application-specific nature of commercial systems. Construction of custom MRgFUS systems is labor-intensive and requires trial and error, and systems must be validated for their application. For example, in the case of thermal therapy, the in vivo response has been shown to be dose dependent [11, 12], particularly in the case of hyperthermia where avoiding the cell death threshold is key, and therapy requires a precise thermal dosage, robust, fine control over the sonication, and accurate thermal monitoring. Developing and debugging a system with these capabilities takes time and expertise which could be a roadblock to researchers who aim to develop new MRgFUS techniques and applications.

In this work, we describe in detail a validated, open-source preclinical MRgFUS platform, with the goal of enabling early-stage MRgFUS researchers to build their own systems with minimal new design and software development effort. The system provides a baseline functionality for performing MRgFUS treatments with inherent flexibility in a modular code structure and freely editable design that can be refashioned for many applications. The system’s open-sourced hardware CAD files will enable researchers to adapt it to their own transducers or magnet geometries and to add features to support their research application. Detailed start-up instructions and commented source code, along with access to sample data sets, make setting up the system straightforward while also leaving room for more sophisticated modifications in the future. The system has been validated in tissue-mimicking phantoms with fiber optic probes and in vivo for thermal treatment of murine tumors. The disseminated package comprises hardware schematics and MR temperature mapping and FUS control software with closed-loop feedback that enables real-time monitoring of the treatment with MR thermometry.

## Methods

### System overview

**Fig. 1:**
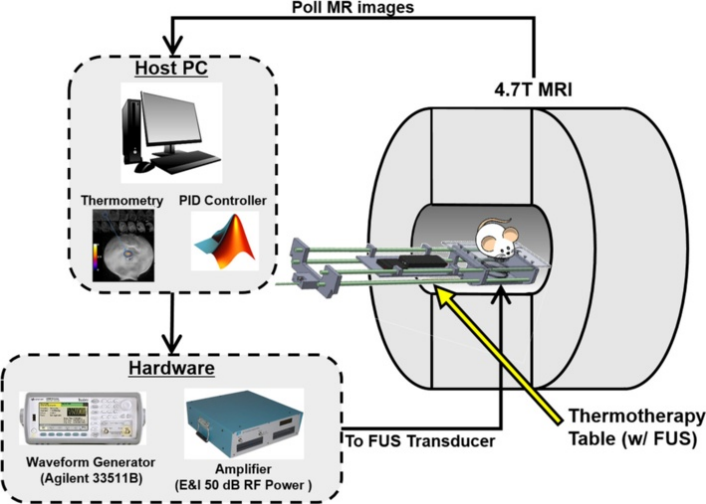
Open-source small-animal MRgFUS system overview. The delivery table holds the target and transducer at magnet isocenter while imaging is performed. Therapy control software for planning and closed-loop temperature control is implemented in Matlab on the MRI scanner’s host PC, which collects the real-time MR images, computes the focal temperature, and modulates the ultrasound output accordingly

All schematics and codes required to construct this system are available for download on GitHub [13]. Figure 1 gives a functional overview of the system. It comprises Matlab-based software (MathWorks Inc., Natick, MA) that runs on the MR scanner host PC and a custom thermotherapy delivery table, built to hold a commercially available FUS transducer within the magnet bore along with associated animal monitoring equipment. Treatment planning involves conventional MR imaging to localize the target tissue above the transducer and parametric analysis of tissue properties. During treatment, an MR thermometry sequence is run continuously and images are read into Matlab in real-time from the scanner file system. MR image phase differences are used to calculate temperature maps within the target tissue and focal temperature is used as input to a proportional integral derivative (PID) controller. The controller computes the required transducer driving voltage to achieve a specified temperature rise in the target tissue based on the temperature evolution over time. The controller output is sent as a command over an Ethernet TCP/IP connection to a function generator and amplifier connected to the FUS transducer, enabling adjustment of sonication intensity in real-time. These components are detailed further in the following sections. The provided distribution includes all control software modules as well as Solidworks (Dassault Systèmes, Waltham, MA, USA) drawings of the delivery table and a parts list of purchased commercial components.

### Hardware

#### Thermotherapy delivery table

**Fig. 2:**
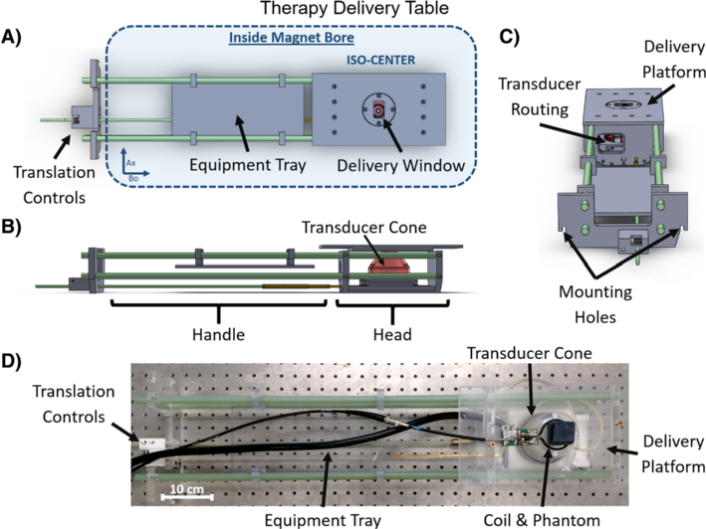
Detailed view of the delivery table. **a** Top view showing placement inside magnet, positioning controls, and rectangular delivery window. **b** Side view showing the housing of the FUS transducer and coupling cone. **c** End view showing routing and mounting locations. **d** Photo of the table to illustrate arrangement of coil and sample

The therapy delivery table comprises an MR-compatible machined Plexiglas fixture with tray and handle that is designed to place the FUS transducer within isocenter for a 21-cm gradient set (Fig. 2). The FUS transducer is secured in place within the head of the delivery table by placing it in a cylindrical slot sized to match its base. Once mounted within the table, the transducer is mechanically positioned using a series of gears. This allows for translation with two degrees of freedom (up to 3.5 cm axial to the magnet’s bore with a rack and pinion and 2 cm in the B0 direction with a lead screw in 1-mm steps) without removing the setup from the magnet bore. Plastic shims can be inserted underneath the transducer to adjust the height of the transducer relative to the platform. Different height coupling cones can be used to adjust the depth of focus. The cone of the transducer is positioned below a 4 cm × 2.5 cm delivery window opening in the platform above it, allowing its direct access to the sample. The delivery window insert can be swapped with windows of varying sizes and shapes depending on the target geometry. An acoustically transparent membrane such as a polymer film can also be stretched over the opening in the platform provided that coupling to the transducer is maintained, though in this work, an open window was found to provide the best coupling and freedom of movement of the transducer. The animal platform measures 15 cm × 28 cm which is large enough to hold a phantom or rodent, associated monitoring equipment, warming pad, anesthesia tube, and RF coil. An imaging RF coil of any configuration can be used and mounted to the platform so long as it does not lie in the path of the ultrasound beam. Holes for FUS power cable routing are integrated in the table, and slots on the end plate are provided for securing the table handle to the front plate of the magnet. A movable tray is attached to the handle of the delivery table to hold any equipment that does not fit on the platform.

#### MR equipment

The therapy table was validated in a Varian 4.7 T preclinical scanner (Agilent, Santa Clara, CA, USA) with a 21-cm bore gradient set (305/210, magnet depth (cm) /inner diameter (mm), Agilent, Santa Clara, CA, USA). All software ran on the scanner’s host PC (Red Hat R5.8, 2.4 GHz Intel Xeon CPU, 12GB RAM). An in-house-built 5-cm diameter Tx/Rx surface coil was used for all imaging and was typically placed flat on the delivery platform between the sample and transducer at the level of the phantom-water interface.

#### Ultrasound equipment

An MR-compatible single element spherically focused ultrasound transducer (Sonic Concepts H101MR, Ellipsoidal full width half max (FWHM): 1.4 mm × 1.4 mm × 10 mm at 1.1 MHz and 0.4 mm × 0.4 mm × 3.2 mm at 3.68 MHz, 400W, Sonic Concepts, Bothell, WA, USA) was used for all validation experiments. The transducer measures 64 mm in diameter with a focal depth of 51.74 mm and was encased in a plastic cone with an open tip for acoustic coupling. Before treatments, the cone was filled with degassed water, the opening was covered with an acoustically transparent latex membrane, and ultrasound gel was applied to couple the cone tip to the sample. Compared to a water or oil-bath immersion approach, this configuration enables easier maintenance of animal core body temperature and the ability to visualize the top of the cone in the MR images for localizing the acoustic focus. The transducer cables extend outside the magnet bore and are connected to the matching network and subsequent amplifier via a BNC cable. The transducer is driven by an Agilent 33511B waveform generator (Agilent, Santa Clara, CA, USA) connected to an E&I RF power amplifier (E&I A150, 150 W, 55 dB, Electronics & Innovation, Ltd., Rochester, NY, USA). The waveform generator is connected via Ethernet to the same network as the MR scanner running the control software, to enable software control of the generator’s output.

### Software

**Fig. 3:**
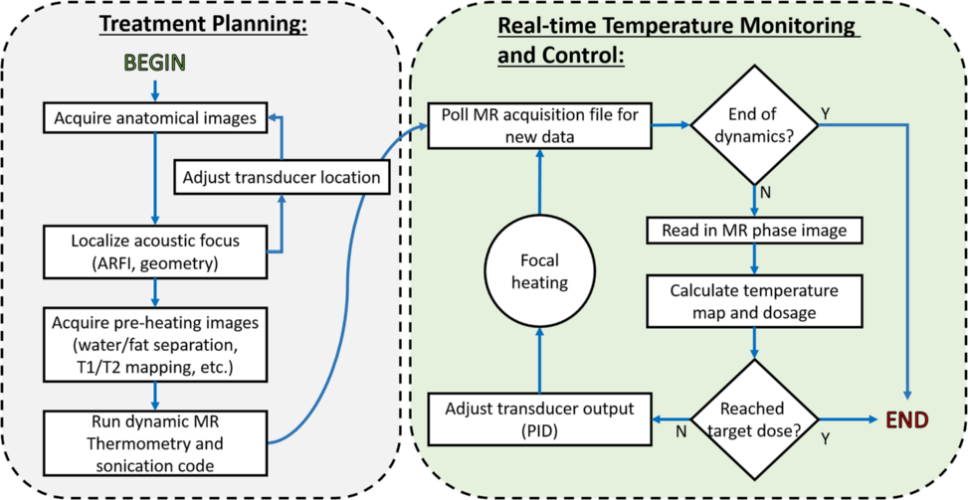
Software flow chart. The treatment protocol comprises a planning stage followed by real-time temperature monitoring and control. The software design allows anatomical and parametric imaging prior to sonication for treatment planning. The temperature monitoring control loop will adjust the FUS amplitude according to observed heating, automatically stopping treatment when a desired thermal dose is achieved

**Fig. 4:**
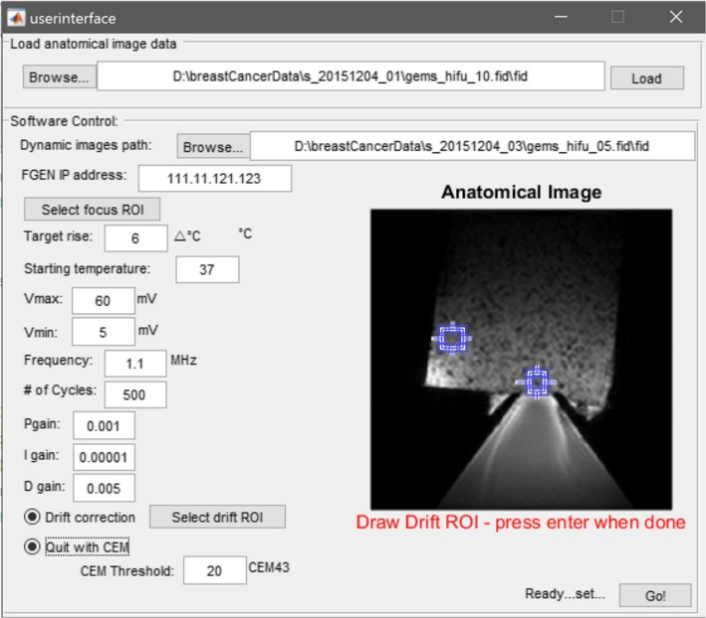
Optional GUI for the setup of the control software. The user can draw ROIs on an anatomical image for the acoustic focus and drift control, set the ultrasound parameters, tune the control parameters, and define a thermal dose target

The user interface and control software was implemented in Matlab and comprises two stages, “Treatment Planning” and “Real-time Temperature Monitoring and Control” (Fig. 3). The code is modular so that elements can be tailored to a specific hardware setup and application while maintaining compatibility with the underlying architecture. These modules, including the function generator initialization, the PID controller, the thermal dose calculation, and data processing, are called from a master script that controls the entire sonication and reconstruction. An optional graphical user interface (GUI) is provided for straightforward treatment planning (Fig. 4). The GUI allows for the user to draw focal and drift correction ROIs on a *T*_2_-weighted anatomical image of the target as well as define a path to the acquisition file and function generator address, controller gains, set a thermal target, toggle drift correction and thermal dose calculations, and set a destination file for the computed temperature maps. These parameters can also be defined manually within the code without using the GUI. After the initial setup, the user is prompted to start the thermometry sequence on the scanner and real-time temperature monitoring and control begins. During treatment, the focal temperature evolution and voltage output over time as well as the latest magnitude image and computed temperature map are displayed for online treatment monitoring.

#### Treatment planning

A suite of MR scan protocols was developed for treatment planning, including anatomical *T*_1_ and *T*_2_ weighted scans, an MR acoustic radiation force imaging (MR-ARFI) scan for focus localization (detailed further below) [14], and a multiple gradient echo scan for water-fat separation (Table 1). An anatomic planning image (usually *T*_2_-weighted) can be imported into the optional user interface to aid in thermometry ROI placement.

**Table 1:** MR imaging sequences and parameters

Sequence	Parameters	Purpose
Gem	TE = 6–15 ms	Gradient echo sequence for PRF-shift thermometry.
	TR = 30 ms	All monitoring was conducted in a single
	Angle = 25	slice in the MRI axial plane, parallel to the direction
	Mat = 96×96	of acoustic propagation. 1–2 dummy
	FOV = 60×60 mm	scans were used to suppress steady-state artifacts.
Fsems	TE = 37 ms	
	TR = 3000 ms	Fast spin echo sequence for *T*_2_-weighted anatomical
	ETL = 8	imaging. Enables tumor localization and visualization
	ESP = 9 ms	of the surrounding environment.
	Angle = 20	
Mgems	TE = 2 ms	
	*Δ*TE = 3 ms	
	TR = 30 ms	Multi-echo gradient echo scan for water/fat separation
	Angle = 25	in post-treatment analysis.
	Mat = 96×96	
	FOV = 60×60 mm	
Gems_meg	TE = 7.6 ms	Modified gradient echo sequence for ARFI. G_dur
	TR = 71 ms	represents the duration a single lobe of the biopolar
	G_amp = 10 G/cm	MEG. The direction of motion encoding was
	G_dur = 4 ms	controlled within the scanner interface based on
	FUS = 1.1 MHz	the slice orientation.

All scans except MR-ARFI were implemented as Varian protocols and did not require new sequence development. The MR-ARFI pulse sequence was implemented based on the Varian “gems” (gradient echo multislice) sequence to visualize the acoustic focus without inducing a significant thermal effect. The source code for the ARFI sequence is in the distributed package. A motion-encoding gradient (MEG) was inserted into a gradient echo sequence immediately following the excitation pulse and prior to the encoding gradients [15]. The MEG parameters such as orientation, duration, shape, and strength are adjusted in the scanner interface to align with the specific geometry of the transducer and target; ARFI encoding is typically performed in the direction of acoustic propagation. The sequence generates a TTL pulse that triggers an ultrasound pulse during the second lobe of the bipolar MEG. Immediately following the MEG, a delay of 1 *μ*s is inserted to prevent gradient overlap before continuing with the spatial encoding gradient waveform. The number of FUS cycles (and thus the length of the pulse) is set on the function generator such that FUS is applied for the duration of the gradient lobe. The sequence is run twice with opposite polarization of the MEG, and the phases of the resulting two images are subtracted. The resulting difference is proportional to the tissue displacement caused by the ultrasound beam, according to:

1 $$ \Delta x = \frac{\Delta \phi}{2 \gamma G l}  $$

where *Δx* is the displacement, *Δϕ* is the phase difference between images with opposite gradients, *γ* is the gyromagnetic ratio, *G* is the MEG strength, and *l* is the length of the MEG. In this equation, the MEG was approximated by a rectangle since trapezoidal gradient pulses with sharp rises were used. The rise time of the MEG with the 21-cm bore 305/210 gradient set was 52 *μ*s for the gradient characteristics used (Table 1), while a typical total MEG duration of 8 ms is used. Residual phase errors due to eddy currents were removed from the acquired ARFI images in post processing by subtracting the phase of two images acquired at each polarization with FUS on and off. Then, the corrected images acquired with opposite polarization of the MEG were subtracted and scaled according to Eq. 1 to obtain the final displacement maps.

#### Real-time temperature monitoring and control

Once all pre-treatment images are acquired and the treatment is planned, the real-time thermometry loop can be executed. This comprises the bulk of the software, informing the ultrasound output directly from images acquired simultaneously on the scanner. Single-slice, baseline-subtracted proton resonance frequency-shift thermometry was implemented using a gradient echo imaging sequence as described in Table 1 with a temporal resolution of 3 s. Scanner field drift correction is imperative for accurate MR thermometry, particularly during hyperthermia treatments where a long sonication time at low power is required [10, 16–18]. To address this, a drift correction routine was implemented using the phase shift in an ROI outside the heated region as a reference. During in vivo sonications, ROI-based drift correction often required the addition of a small tube of water to the imaging plane to serve as a reference no-heat region in case the mouse anatomy was too small for a reliable ROI correction. Once the real-time monitoring loop is initialized, the software continuously polls the MR raw data file for new data. To prevent constant file opening and closing that could delay execution, the software only opens the file when the time stamp has changed, meaning a new image has been acquired. One to two dummy scans are acquired prior to the first baseline to prevent steady-state artifacts. Then, the first image acquired in the loop is used as a baseline and subsequent images are used to compute a temperature map relative to the baseline. A focal mean temperature is estimated from the current temperature map and stored. If desired, drift correction is applied at this step to account for scanner drift and thermal dose is computed in CEM43 units [12].

The corrected mean focal temperature along with the current function generator voltage *V*_out_ is then input to a PID controller function along with the desired temperature rise, the PID gain constants, the maximum voltage output limit, and the previous error up to the current dynamic. The function calculates the new *V*_out_ to achieve the desired temperature rise according to:

2 $$ V_{\text{out}} = \text{min}{\left\{K_{\mathrm{p}} e(t) + K_{\mathrm{i}} {\int_{0}^{t}}e(\tau)d\tau + K_{\mathrm{d}} \frac{de}{dt}, V_{\text{max}}\right\}},  $$

where *K*_p_, *K*_i_, *K*_d_ are the proportional, integral, and derivative gain, respectively, *e*(*τ*) is the error between the current temperature and desired temperature, and *t* is the time elapsed since starting sonication. The maximum voltage constraint *V*_max_ is set to maintain the acoustic pressure below the threshold for cavitation during in vivo experiments and minimize skin burns. It also prevents the transmitted power from damaging the transducer. A maximum voltage of 70 mV (prior to 55-dB amplification) was used for all in vivo experiments, corresponding to a peak negative pressure of approximately 1.5 MPa at 1.1 MHz as measured by a ceramic needle hydrophone (HNC-0200, Onda, Sunnyvale, CA). PID gain values are critically important in controlling the behavior of the system and temperature rise at the focus. These gains were manually tuned in a graphite-agar phantom to prevent target temperature overshoot of greater than 1 °C and a steady-state temperature variation of no more than 0.5 °C. The resulting values were: *K*_p_=10^−3^, *K*_i_=10^−5^s/repeat, and *K*_d_=5×10^−3^ s. Once calculated, *V*_out_ is returned to the real-time loop. The software then checks if the measured thermal dose is greater than the defined thermal dose threshold and sets the output to *V*_out_=0 if the threshold has been met, turning off the transducer output. The final *V*_out_ is then output to the function generator. If the MR imaging is complete, the loop exits and treatment is halted. Otherwise, the loop repeats, modulating the transducer output to maintain a precise and accurate temperature rise within the target for the duration of the scan time. In the event of a system failure, the code automatically exits and stops output from the function generator. All MR images were obtained with the parameters listed in Table 1.

### Experiments

#### Fiber optic thermometry validation

**Fig. 5:**
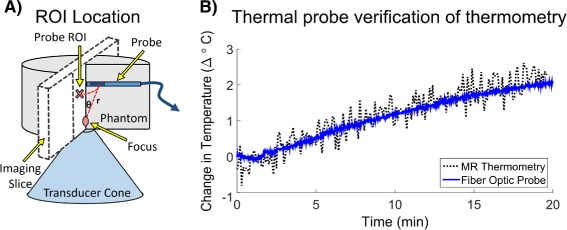
Fiber optic probe thermometry validation. **a** Illustration of the experimental setup. To avoid artifacts and damage to the probe, it was placed above the focus. **b** Plots probe temperature compared to MR temperature measurements in a 5.7 mm^2^ ROI at a geometrically equivalent position within the slice

A graphite-agar phantom (1.5 % agar, 4 % graphite, weight per volume of water [19]) was used to mimic tissue acoustic properties. The phantom was set up on the system and coupled to the transducer cone with ultrasound gel. Prior to sonication, a fiber optic temperature probe (FISO Technologies Inc., Quebec, Canada) was inserted into the phantom just outside of the acoustic focus. The entire setup was placed in the magnet and closed-loop feedback sonication was performed for 20 min under thermal monitoring with a gradient echo thermometry sequence (Table 1). The imaging slice was 3-mm thick and oriented to avoid imaging artifacts due to the heating of the fiber optic probe tip. Probe placement relative to the focus and the location of the imaging slice are illustrated in Fig. 5a. Given the uniformity of the phantom and radial symmetry of the acoustic focus, an ROI that was radially symmetric to the fiber optic probe tip’s location with respect to the focus was chosen within the imaging slice for the mean temperature calculation.

#### Constant temperature control validation

To validate the closed-loop control software, a graphite-agar phantom was again placed on the delivery platform, coupled to the transducer and placed within the magnet. Five sonications lasting 10 min each were conducted with the system at target temperature rises between 2 and 10 °C. A single 3-mm thick axial slice through the acoustic focus was used for thermal monitoring. The phantom was allowed to cool for 2 min between each sonication, and the PID gain values remained fixed throughout. For all closed-loop experiments, precision and accuracy measures of the temperature rise were calculated from the initial temperature rise, defined as the point at which the mean focal temperature first crossed the set temperature threshold.

#### Closed-loop feedback at two FUS frequencies

Raw chicken and graphite-agar phantoms were used to validate the closed-loop feedback sonication at the transducer’s two operating frequencies (1.1 and 3.68 MHz). In each sonication, a single 3 mm thick axial slice through the acoustic focus was used for thermal monitoring and ROI-based drift correction was performed by placing an ROI in areas of the phantoms that would see negligible heating. The operating frequency was set using the control software and matching network connected to the transducer.

#### In vivo murine tumor treatment

The thermal monitoring and closed-loop feedback system was tested in vivo in a Polyoma PyVMT murine breast cancer tumor model [20] under an approved Institutional Animal Care and Use Committee protocol (M/13/010). This animal model spontaneously generates superficial tumors in the mammary fat pads with a progression comparable to human breast cancer. Tumors measuring ≤1 cm in diameter and located most distal to the lungs were chosen for targeting with FUS in order to minimize breathing artifacts. Fur in the treatment area was removed with depilatory cream prior to treatment for improved acoustic coupling. The animal breathing rate was maintained throughout around 60 breaths per minute with isoflurane anesthesia ranging from 1.5 to 2.5 %. The tumor was coupled to the transducer cone with ultrasound gel, and core body temperature was maintained with a circulating hot water pad. Localized hyperthermia was applied with the control software under thermal monitoring in a 3-mm-thick axial slice through the focus at 1.1 MHz for 12 min. No drift correction was applied for this mouse although both a lookup table method, with precalculated drift compensation, and roi-based correction method have been used successfully with this system. The calculated focal temperature and PID controller output were observed to characterize the system behavior.

#### Transducer translation validation

The system was used to deliver four ablative sonications to a polyacrylamide gel phantom containing egg white [21]. The phantom was designed to be translucent except in areas of heating where the egg white would coagulate. Ablative treatments were manually applied for 2 min at a peak negative pressure of 3.9 MPa, without temperature feedback. Between sonications, the transducer was translated in the slice plane using the translation controls outside of the magnet and positioning was confirmed with *T*_1_-weighted images visualizing the water-filled transducer cone and the sample. After all sonications were completed, a *T*_2_-weighted image was acquired and a photograph was taken of the coagulated egg white lesions visible in the phantom. The distances between the lesions were calculated using both images and compared to assess relative position accuracy.

#### Mechanical displacement with ARFI

MR-ARFI measurements were made in a tofu phantom that was coupled to a short transducer cone to increase the penetration depth of the transducer and enable visualization of the near and far fields of the focus within the phantom. ARFI images were acquired in an axial and coronal slice centered around the acoustic focus at 1.1 MHz with a 2.5-MPa peak negative pressure (5.6 % duty cycle). Optimal coronal slice placement was determined by acquiring ARFI images across the entire phantom and choosing the slice of most localized displacement, indicating a position at the focus. Axial placement was confirmed by centering the slice over the transducer water cone visible in the anatomical images. For each slice orientation, the motion-encoding gradients were oriented in the direction of acoustic propagation.

## Results

### Fiber optic thermometry validation

Figure 5b shows a comparison of the temperature measured during sonication with MR thermometry and the fiber optic probe. The mean temperature recorded with MR thermometry in the 5.7 mm^2^ equivalent ROI was accurate relative to the thermal probe with an RMSE over time of 0.07 °C and maximum error less than 1 °C. The thermometry measurements were noisier than the probe measurements but had an acceptable level of precision with a standard error of 0.25 °C.

### System behavior at varied target temperatures

**Fig. 6:**
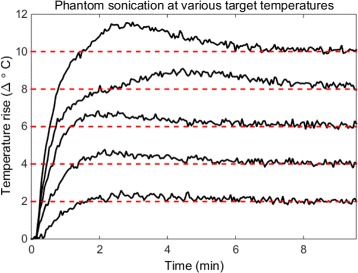
Sonications across temperature set points. After initial overshoots that did not exceed 1.5 °C of the set points (*dashed red lines*), focal temperature was maintained for 10 min with a mean standard deviation of the temperature error of 0.28 °C and a mean RMSE of 0.3 °C

For all sonications, no lag in software execution was observed. The control software run on the scanner computer executed fully within the 3-s time frame of each image as detailed in Table 2. Figure 6 plots the mean focal temperature in a phantom subjected to multiple sonications at set points ranging from 2 to 10 °C. The focal ROI used to calculate the mean temperature was 2.6 mm × 3.2 mm which encompasses the full width half max of the transducer’s focus. In each case the temperature reached a steady state around the desired temperature within a few minutes, with an initial overshoot of less than 1 °C except for the 10 °C sonication which had an initial overshoot less than 1.5 °C. After the initial temperature rise, the mean standard deviation of the temperature error was 0.28 °C with a mean RMSE of 0.44 °C.

**Table 2:** Execution speed of the real-time software

Action	Mean execution time (ms)	Purpose
Initialize function generator	1885 ms	Performed once before each temperature-controlled sonication, this action opens communication between the host PC and the ultrasound function generator and configures the function generator with the desired output parameters for sonication.
Read in image	42 ms	Time to open the raw MR data (.fid) file and reconstruct the magnitude and phase data into an image for thermometry.
Compute temperature map	39 ms	Time to construct a temperature map with baseline subtraction of image phases after new data has been read. This timing includes drift correction with subtraction of phase from a reference ROI.
Output voltage to function generator	1 ms	Time to evaluate PID equation based on current focal temperature and system state and send *V*_out_ to the function generator.

### Closed-loop feedback at two FUS frequencies

**Fig. 7:**
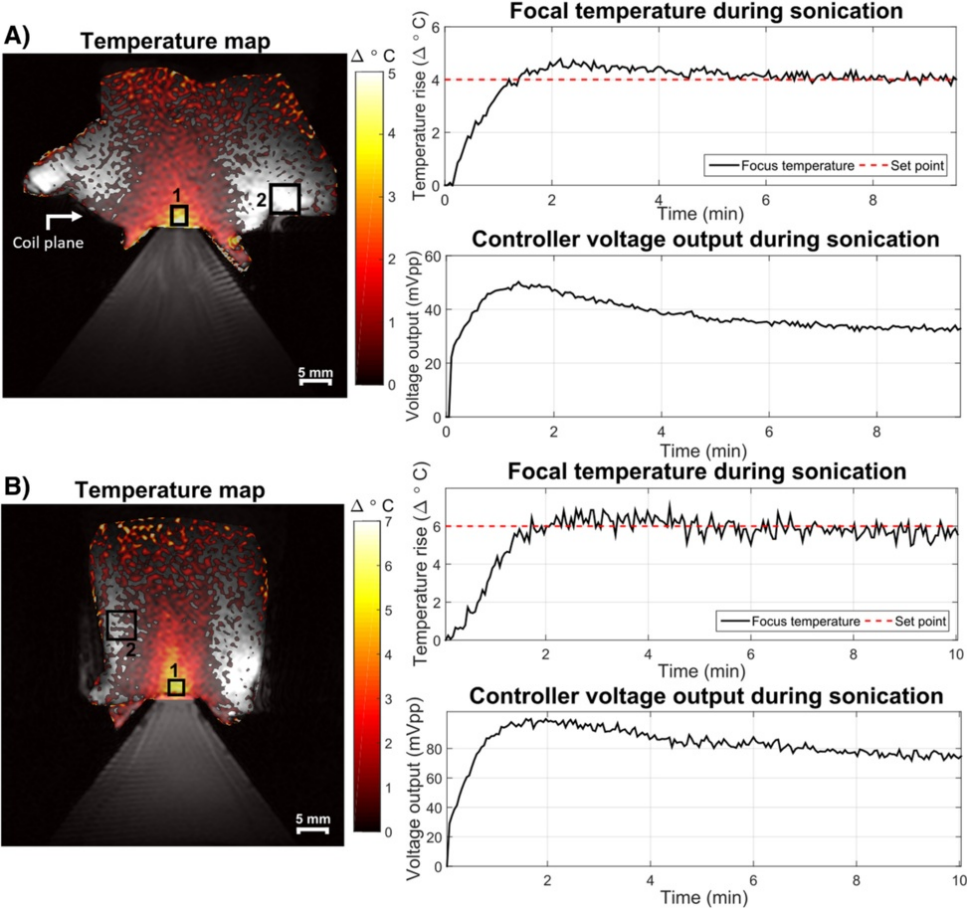
Sonications at 1.1 (**a**) and 3.68 (**b**) MHz FUS frequencies, targeting temperature set points in ROI 1 for 10 min. Background phase drifts were corrected using an ROI outside of the area of heating (ROI 2). Controller voltage is also plotted for each case and also stabilizes after an initial rise and small overshoot. The *white arrow* indicates surface coil placement. Low-temperature SNR at the top of the phantoms (and far from the surface coil which sat at the level of the water-phantom interface) contributed to the apparent elevated temperatures there but did not interfere with the focus measurements. Stripe artifacts in the water cone are likely due to Moire fringes caused by poor field homogeneity in the water bath near the transducer causing aliasing

Figure 7 shows the sonication of two phantoms at 1.1 (a) and 3.68 (b) MHz FUS frequencies. The left side of the figure shows representative treatment temperature maps overlaid on a *T*_1_-weighted image of the phantom (the baseline thermometry image). The right side plots the mean focal temperature over time as measured by MR thermometry and the commanded function generator voltage *V*_out_. The focal ROIs used for the mean temperature calculation were 2.6 mm × 3.2 mm at 1.1 MHz and 2.6 mm × 2.6 mm at 3.68 MHz. The ROIs used for drift correction are also displayed in the figure and were each 4.6 mm × 4.6 mm. Temperature overshoot in each case was less than 1 °C with the standard deviations of the errors measured to be 0.21 °C and 0.43 °C at 1.1 and 3.68 MHz, respectively. After the initial overshoot, the RMSE of the mean temperature measured was 0.31 °C for the 1.1 MHz sonication and 0.61 °C for the 3.68 MHz sonication. A steady state was achieved within a few minutes, as noted by the leveling off in the voltage output over time.

### In vivo murine tumor treatment

**Fig. 8:**
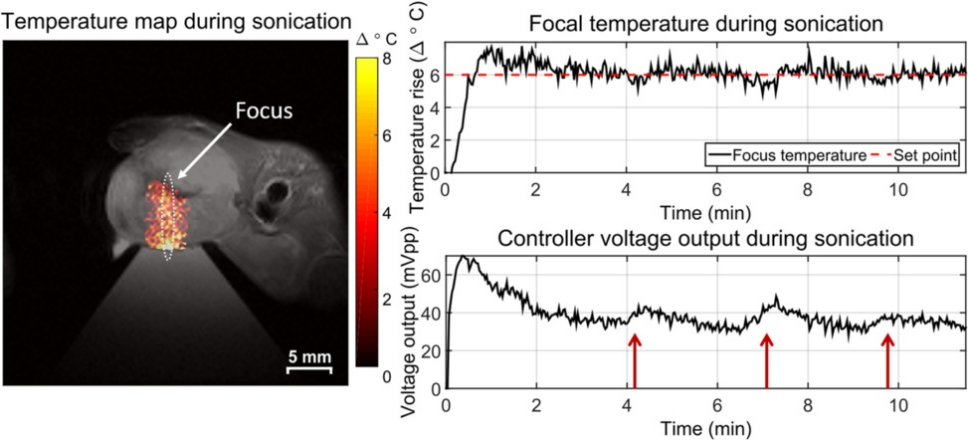
Sustained local hyperthermia in a murine mammary tumor for 12 min. Sustained, long-term sonication was achieved with minimal overshoot, a 0.49 °C standard deviation of the error, and 0.56 °C RMSE after the initial temperature rise. The PID controller responded to sudden changes in focal temperature as indicated by the *red arrows*. The 1.4 mm by 10 mm contour of the transducer focus is indicated by the *white oval*

Figure 8 shows an in vivo sonication of a murine mammary tumor treated at *Δ*6 °C for approximately 12 min. On the left, a representative temperature map during treatment is overlaid onto a *T*_2_-weighted anatomical image of the mouse. The focal ROI size was 2.5 mm × 2.5 mm. The two curves on the right show the mean focal temperature evolution over time and the corresponding peak-to-peak voltage output from the PID controller to the transducer. After an initial overshoot of less than 1.5 °C, the focal temperature reached a steady state (noted again by the leveling off of the voltage output with time) with some variations. Three major dips in the mean temperature reading and subsequent bumps in the voltage output occur around 4, 7, and 10 min as noted by the red arrows in the figure. These perturbations corresponded with times when the mouse started breathing at a faster rate as observed by the monitoring equipment. The PID controller responded appropriately by increasing the voltage output when a sudden decrease in temperature was observed. The controller was able to compensate for the change in conditions and maintain the temperature at the set point with a 0.49 °C standard deviation of the error and 0.53 °C RMSE after the initial temperature rise.

### Transducer translation

**Fig. 9:**
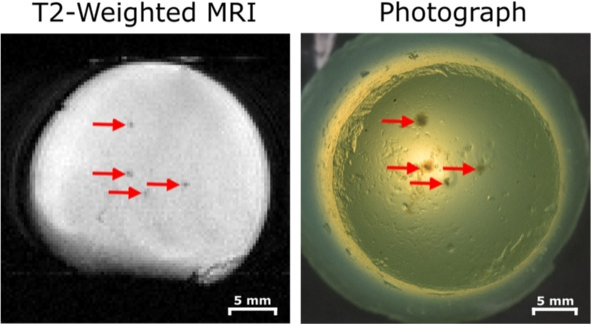
Demonstration of transducer translation capabilities via multiple egg white phantom ablations. The transducer was moved using the controls outside of the magnet; no re-positioning of the phantom or removal of the platform was necessary. Lesions were visible on a *T*_2_-weighted image (*left*) and on photographs taken outside the magnet (*right*) and matched geometrically

Figure 9 shows a *T*_2_-weighted image and photograph after four ablative sonications were performed in an acrylamide-albumin phantom. No removal of the setup from the magnet was required to move the transducer. The ablated lesions are clearly visible on the *T*_2_-weighted image and qualitatively line up well with the coagulated egg white lesions visible in the photograph. The mean error in the distance between lesions in the two images was found to be 0.11 mm demonstrating good relative positioning accuracy.

### Mechanical displacement with ARFI

**Fig. 10:**
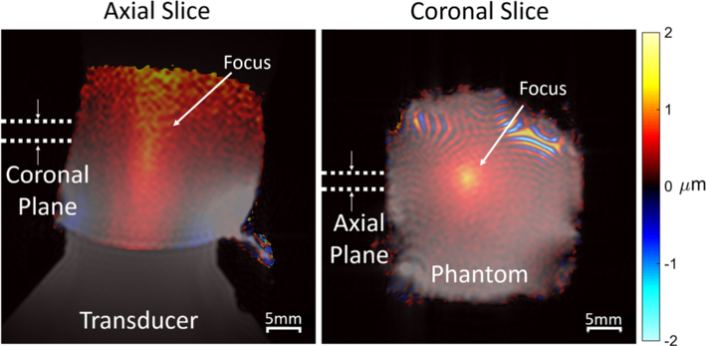
ARFI-measured displacement in the axial and coronal directions overlaid on *T*_1_-weighted images of a tofu phantom. Localized displacements are apparent at the focus while smoother displacements appear throughout which may correspond to shear waves. The focal displacement location and size correspond well to the expected geometry of the transducer. Acoustic reflections with the air boundary and lower SNR near the top of the phantom likely contribute to the diffuse rise in displacement there. Moire fringes are visible at the top of the coronal image, which are likely due to field inhomogeneity near the surface of the transducer causing some water bath signals to be excited and alias into the image

Figure 10 shows the axial and coronal displacement of the tofu phantom with applied FUS as imaged with the ARFI sequence. The displacement maps were overlaid on *T*_1_-weighted magnitude images for visualization. The measured displacement was consistent between slice orientations, with maximum displacements of 1.0 *μ*m (axial) and 1.2 *μ*m (coronal). The FWHM for the axial and coronal maps were 3.0 and 2.93 mm, respectively, which are comparable to the 1.4-mm FWHM intensity profile of the transducer but may be broader in this measurement due to phantom mechanical properties and shear waves.

## Discussion

### Summary of results

We have successfully designed, constructed, and tested an open-source preclinical MRgFUS hardware and software package in phantoms and in vivo. The system was shown to be capable of robustly sustaining controlled temperature rises with MRgFUS in a preclinical setting. The platform is MRI compatible, allowing for unobstructed imaging and sonication of the target with two degrees of freedom in transducer motion and minimal removal of the hardware once placed. Treatment planning tools were implemented, comprising standard anatomical scans and an MR-ARFI sequence to image mechanical displacement due to FUS. The real-time temperature mapping and drift correction routine was shown to be accurate to within 0.07 °C when compared to a fiber optic thermal probe reference and was able to complete computations online within one image frame. The use of a 3-mm slice thickness during thermometry, which was larger than the focus width and chosen to increase image SNR, could contribute to the initial temperature underestimation within the focus [22]. However, with the long sonication duration and thermal diffusion in hyperthermia, we do not expect this to be a problem. For shorter treatments such as ablations, users should choose their slice thickness accordingly to prevent underestimation. A steady state was achieved for all sonications during which the PID control software responded appropriately to changing conditions in vivo and maintained the desired temperature rise for the entire duration of treatment. These characteristics make the described system viable for use in studies with MRgFUS on small-animal models.

We expect minimal training to be necessary for new users. Two undergraduate researchers in our group learned to setup and run the system with 5 h of training. The entire system was constructed for less than $20,000 USD of which approximately $600 USD was spent designing and constructing the delivery table. Many of the commercially purchased products, such as the amplifier, were intentionally purchased with specifications far exceeding those required for this system. Further reduction of cost could be achieved depending on the hardware specifications required by the application and preexisting availability of such equipment to the user.

### Steady state with PID control

The implemented PID controller allowed the system to robustly maintain the focal temperature rise at a desired set point without any visible effect from the scanner’s field drift or transducer frequency used. The controller was responsive to changes in perfusion during in vivo heating [23], as evidenced by upticks in the output voltage in Fig. 8; however, proper tuning of the PID controller gains is important for controlling the response of the system in such situations. The PID controller performance has been shown previously to be robust to noise [24]; however, adjusting the integral gain of the controller might also improve noise stability [25]. For this study, the PID parameters were tuned once manually in a tissue-mimicking phantom and remained constant during all experiments described. This approach was sufficient for our purposes as the temperature set points and tissue properties did not vary significantly between experiments. This may also be the case for many hyperthermic applications where a set point of *Δ*6 °C from baseline (or 43 °C) is desired and does not vary significantly between experiments. Individual tuning may also be avoided for studies that target tissues with similar properties between subjects. These settings have been used successfully in four more mice with no observed disruption from the PID controller settings [26]. However, if a study were to involve more heterogeneous tissue or largely varied target temperature rises, the controller gains would likely need to be tuned for each specific tissue and set point. Controller gains would likely also be required for different applications, such as hyperthermia vs ablation where overshoot of the target could severely impact the experimental outcome. In these cases, the user could alter the PID controller’s behavior by adjusting the PID gain values. For example, the early temperature overshoot we observed in some of our sonications could be reduced by increasing the derivative gain with a potential trade off of a slower start-up. These values can be freely adjusted by the user within the GUI or software code to tune the controller output. Tuning could be performed using previously described algorithms [27, 28].

### Temporal resolution

For real-time monitoring, a fine temporal resolution is desirable to maintain tight control of heating and rapidly detect unintended heating [10]. In addition to MR acquisition time, another main factor that could limit the temporal resolution of this system is the execution speed of the real-time software. This is highly dependent on the computer specifications; however, care was taken in the code development to minimize execution time. For example, memory is preallocated for large variables and the MR raw data file is opened only when the time stamp on the data has changed from the previous check. This prevents the file from being continually opened and closed extraneously, avoiding any associated lag time. For the software run on the scanner computer, execution speeds were as detailed in Table 2. The software executed in less than a second after the inital setup which is shorter than the thermometry sequence’s temporal resolution of 3 s with no observed delay in temperature mapping. In the case that a shorter temporal resolution were needed, an accelerated image acquisition scheme such as EPI [29] or partial Fourier [30] could be implemented. The operation of the system should remain the same provided that the software execution time does not exceed the time required to acquire the next image.

### Contemporary systems

Other research groups have also developed systems to meet the challenge of treating small-animal models with MRgFUS; yet, there are underlying differences that make our system unique. One such system was recently described by Bing et al. [31], which was also based on a constrained PID controller for fine control over the focal temperature rise in vivo. The Bing system was designed for a 3-T human MRI scanner and based on a commercial clinical MRgFUS system (RK100, FUS Instruments, Toronto, Canada), which are of more limited availability and much higher cost than the described system. Our system was intended for use with small-animal MRI scanners more commonly used in preclinical research and has the flexibility with open-source CAD plans to be adapted to use with scanners of many configurations. Many of the current hardware components were machined by hand but could be 3D printed provided that the material used was strong enough to remain structurally sound with use. An interesting development in Ref. [31] was the use of acoustic lenses to diffuse the focus and deliver heat over a larger volume. For the mouse model used in our experiments, the 1.4 × 10 mm focal size of our transducer at 1.1 MHz was sufficient; however, other applications could benefit from larger focal sizes enabled by such lenses. In this case, it would be possible to adapt the transducer cone used with our system to include an acoustic lens.

Another contemporary system was described by Fite et al. [27]. In their system, MRgFUS was also implemented on a preclinical MRI scanner; however, commercial hardware was used to equip the magnet with FUS capabilities (Thermoguide, Image Guided Therapy, Pessac, France) leading to cost and design flexibility limitations similar to Bing et al. The Fite system used a PID controller tuning algorithm using the Pennes Bioheat Equation [32] that enabled exact control characteristics to be easily achieved. As mentioned previously, such tuning algorithms could be integrated with our system to avoid manual tuning of the PID controller. One feature of the Fite system is the integrated quadrature coil that provides high sensitivity throughout the target volume during treatment. Our system uses a surface coil which provides high sensitivity at the focus level but has less sensitivity further into the volume. This sensitivity gradient does not interfere with temperature visualization in our system; however, image SNR could be further improved by adaptation of a quadrature coil similar to the Fite system. We found that our temperature measurements were accurate to within 0.07 °C, and precise with a standard error of 0.25 °C, which is comparable with that of the commercial system used by Fite et al. (Ref. [27], Figure 4) as well as clinical systems such as the Philips Sonalleve (Philips Healthcare, Best, Netherlands), for which an accuracy within ±1 °C is reported [33].

An additional system was described by Magnin et al. [34]. Like our system and the Fite system, the Magnin system was designed for a preclinical MRI scanner. However, the FUS control software used by Magnin was commercially purchased (Thermoguide, Image Guided Therapy, Pessac, France). The main feature that sets their implementation apart is the use of a motorized frame to adjust the transducer position. The Magnin system allows for translation in three dimensions as well as electronic steering with multiple transducer elements while ours has two degrees of freedom and uses a single element transducer. This freedom of motion works very well for their proposed application in transcranial FUS. It should be noted that the transducer used in the Magnin system was much smaller than the one implemented in our system. As such, the exact motorized framework used by Magnin may not be usable with our larger transducer size, although a similar motorized design could be adapted. For the hyperthermic application explored in this paper, our system’s translation and steering capabilities were sufficient.

Finally, while our system’s most fundamental features such as treatment planning and monitoring software, real-time MR thermometry-based closed-loop temperature control, and an MR-compatible therapy table parallel the features of a clinical MRgFUS system, the current clinical systems now include more sophisticated features such as active transducer cooling, active skin cooling, and electronic and mechanical beam steering. While our system does not currently have these features, the underlying framework is comparable and inherently allows for more flexibility of design and application-specific modification than a more regulated clinical system might provide.

### Possible extensions

The open-source nature of the system allows it to be adapted to the specific equipment requirements of the group using the system. The current delivery platform with flat top and modular delivery window provides inherent flexibility in target placement; however, this can make the experimental setup challenging. During experiments, positioning the coil for good SNR while not interfering with the acoustic coupling, the circulating water heating pad (Kent Scientific, Torrington, CT, USA) or the animal monitoring equipment required the use of tape and foam supports. These considerations led to relatively long setup times, particularly for in vivo experiments where it often took up to 25 min to position the mouse, tune and match the coil, and calibrate the MR scanner for imaging. Such challenges could be mitigated by the use of a mouse holder that screws into the platform, provides mechanical support, connects to anesthesia and monitoring equipment, and maintains body temperature. In addition, the current single-slice baseline subtracted thermometry routine was prone to susceptibility artifacts from gut motion, respiration, or the presence of fat depending on the imaging slice. This could be improved through the implementation of more motion-robust temperature reconstruction, which is an open area of research [35–46]; given an effective approach to this problem, the associated processing could be incorporated in our modular processing framework. Additional modules could be added by the user to incorporate other feedback modes (i.e., concurrent ARFI imaging, passive cavitation detection) as well as making the required MR pulse sequence or hardware changes. Hardware modifications can be made in the provided Solidworks designs prior to construction. For example, the delivery table could be modified to hold transducers of other geometries by changing the size or shape of the cylindrical slot within the head of the table that holds the transducer. The length, width, and height of the delivery table could be adjusted to accomodate MR scanners of different bore sizes provided that the ultrasound transducer still fits within the table. If the addition of plastic shims does not provide enough freedom of adjustment in the transducer height, a second rack and pinion system could be constructed to allow the transducer to be raised and lowered relatively to the platform from outside of the magnet. The open-source files and modular structure of the software are intended to make such adaptations straightforward for users to implement.

## Conclusions

We have described and validated a preclinical closed-loop MRgFUS system. Defined completely in open-source Matlab and Solidworks files, we hope to lower the initial barrier to conducting small-animal MRgFUS studies. The described system represents a cost-effective solution that allows for flexibility in design and implementation to suit the needs of cross-disciplinary researchers in conducting preclinical studies with FUS.

## Abbreviations

ARFI: Acoustic radiation force imaging
MEG: Motion encoding gradient
MRgFUS: Magnetic resonance-guided focused ultrasound
PID: Proportional integral derivative; PRF Proton resonant frequency
