# Erratum to: Open-source, small-animal magnetic resonance-guided focused ultrasound system

**DOI:** 10.1186/s40349-016-0070-y

**Published:** 2016-10-19

**Authors:** Megan E. Poorman, Vandiver L. Chaplin, Ken Wilkens, Mary D. Dockery, Todd D. Giorgio, William A. Grissom, Charles F. Caskey

**Affiliations:** 1 Department of Biomedical Engineering, Vanderbilt University, PMB 351631 2301 Vanderbilt Place, Nashville, TN 37235 USA; 2 Institute of Imaging Science, Vanderbilt University, 1161 21st Avenue South, Nashville, TN 37232 USA; 3 Chemical and Physical Biology Program, Vanderbilt University, 1161 21st Avenue South, Nashville, TN 37232 USA; 4 Department of Radiology, Vanderbilt University, 1161 21st Avenue South, Nashville, TN 37232 USA

## Erratum

In the publication of this article [1] it was brought to our attention that one of the funding sources was incorrectly presented as DoD W81XW-12-BCRP-IDEA. This number is actually the granting mechanism and not the specific grant number.

The correct grant number should read W81XWH-13-1-0230, which is under the DoD BCRP-IDEA umbrella and should be the one mentioned in the Declaration section of this article.
